# Pneumococcal meningitis: Clinical-pathological correlations (meningene-path)

**DOI:** 10.1186/s40478-016-0297-4

**Published:** 2016-03-22

**Authors:** Joo-Yeon Engelen-Lee, Matthijs C. Brouwer, Eleonora Aronica, Diederik van de Beek

**Affiliations:** Department of Neurology, Academic Medical Center, University of Amsterdam, Center of Infection and Immunity Amsterdam (CINIMA), PO Box 22660, 1100DD Amsterdam, The Netherlands; Department of Neurology, Academic Medical Center, University of Amsterdam, Amsterdam, The Netherlands; Swammerdam Institute for Life Sciences, Center for Neuroscience, University of Amsterdam, Amsterdam, The Netherlands

**Keywords:** Pneumococcal meningitis, Histopathology, Vascular inflammation, Apoptosis

## Abstract

Pneumococcal meningitis is associated with substantial mortality and morbidity. We systematically assessed brain histopathology of 31 patients who died of pneumococcal meningitis from a nationwide study (median age 67 years; 21 (67 %) were male) using a pathology score including inflammation and vascular damage. Of the 27 patients with known time from the admission to death, 14 patients died within 7 days of admission and 13 after 7 days of admission. Eleven of 25 (44 %) patients had been treated with adjunctive dexamethasone therapy. Observed pathological processes were inflammation of medium-large arteries in 30 brains (97 %), cerebral haemorrhage in 24 (77 %), cerebritis in 24 (77 %), thrombosis in 21 (68 %), infarction in 19 (61 %) and ventriculitis in 19 (of 28 cases, 68 %). Inflammation of medium-large arteries led to obstruction of the vascular lumen in 14 (of 31 cases, 45 %). Vascular inflammation was associated with infarction and thrombosis of brain parenchymal vessels. Hippocampal dentate gyrus apoptosis between patients treated with and without dexamethasone was similar (*p* = 0.66); however, dexamethasone treated patients had higher total pathology score than non-dexamethasone treated patients (*p* = 0.003). Our study shows that vascular damage is key in the process of brain damage in pneumococcal meningitis. Data and material of this study will be made open-access for translational research in pneumococcal meningitis (MeninGene-Path).

**Electronic supplementary material:**

The online version of this article (doi:10.1186/s40478-016-0297-4) contains supplementary material, which is available to authorized users.

## Introduction

Bacterial meningitis is an infection of the central nervous system with an incidence of 0.9–2.6 per 100.000 per year in adults in high-income countries and substantially higher incidences in countries with middle- and low-income status [1, 2]. *Streptococcus pneumoniae* (the pneumococcus) is the most frequent causative micro-organism of community-acquired bacterial meningitis, causing 70 % of adult cases [1, 2]. Pneumococcal meningitis is associated with high mortality and morbidity rates, with 18–30 % of patients dying, and neurological sequelae occurring in half of survivors, most commonly hearing loss and cognitive deficits [3–6]. The poor prognosis of pneumococcal meningitis has been hypothesized to be driven by a high rate of cerebrovascular complications, mainly consisting of cerebral infarctions [7].

Few studies have systematically analysed brain autopsy material from patients with pneumococcal meningitis [8–10]. These studies showed that parenchymal damage in pneumococcal meningitis is caused by the combination of cytotoxic and vasogenic oedema, compression of vital brain structures, leukocyte infiltration, abscess formation, and cortical necrosis [8–10]. Animal models showed that hippocampal apoptosis was associated with abnormal neuropsychological test results in animals after pneumococcal meningitis, and that poor neuropsychological test results were associated with adjunctive dexamethasone treatment [9, 11]. Experimental animal models of pneumococcal meningitis furthermore have shown a large variation in histopathological features, most likely due to different combinations of bacterial strains, infected animal species, methods of inoculation, and stages of infection [12–18].

Adjunctive dexamethasone is currently routine in the treatment of adults with community-acquired pneumococcal meningitis after evidence from randomized clinical trial [3], meta-analyses [19–20], and nationwide implementation studies [21, 22]. Although dexamethasone has been associated with increase hippocampal apoptosis in mice with pneumococcal meningitis [9, 11], a long-term follow-up study of a randomized controlled trial investigating the efficacy and safety of adjunctive dexamethasone did not show increased neuropsychological sequelae in those treated with dexamethasone [6].

Histopathological examination may provide insight in pathophysiological mechanisms that take place during pneumococcal meningitis [23]. In 2006, we started a prospective cohort study to identify and characterize host genetic traits and bacterial genetic factors controlling occurrence and outcome of bacterial meningitis (MeninGene) [24]. Previously, we reported the incidence, causative pathogens, clinical features, and prognostic factors in adults with community-acquired bacterial meningitis in the Netherlands from 2006 to 2014 [21]. Here, we report on the systematic scoring of histopathology material of 31 pneumococcal meningitis cases.

## Materials and methods

### Patients

We identified patients with community-acquired pneumococcal meningitis in whom autopsy was performed between 1985 and 2013 in the neuropathology database of the Academic Medical Center, Amsterdam, and from two nation-wide prospective cohort studies [3]. Methods of both studies have been described previously [3, 22]. In summary, all patients over 16 years of age or older who were listed in the database of the Netherlands Reference Laboratory for Bacterial Meningitis [22] were prospectively included in these cohort studies. This laboratory receives cerebrospinal fluid (CSF) isolates from 90 % of all patients with bacterial meningitis in the Netherlands. The NRLBM provided daily updates of the names of the hospitals where patients with bacterial meningitis had been admitted in the preceding 2–6 days and the names of physicians. Physicians were contacted, and informed consent was obtained from all participating patients or their legally authorized representatives. From the databases of these cohorts we selected deceased patients who underwent brain autopsy. Cases with negative CSF and blood cultures, trauma and/or neurosurgery prior to meningitis were excluded. If the brain autopsy was performed in a hospital other than the AMC, histology slides, tissue blocks and autopsy reports were requested. Informed consent was obtained for brain autopsy. Tissue was obtained and used in accordance with the Declaration of Helsinki and the AMC Research Code.

Brains of twenty-one patients served as age, sex and duration of hospital admission matched control samples for the analysis on apoptosis in the hippocampal cortex. These patients died from non-neurologic disease and were not found to have neurological disease during life. The causes of the death of the controls were: ten cases of sepsis with the admission duration of 1 to 20 days, five cases of cardiovascular accidents with the admission duration of 1 and 17 days, four cases of lung embolism with duration of 1 and 6 days and one case of respiratory failure from an acute exacerbation of end stage chronic obstructive pulmonary disease of which admission duration was unsure. The neuropathologist was blinded for the patient diagnosis at the time the brains were re-evaluated.

### Histopathology

**Fig. 1:**
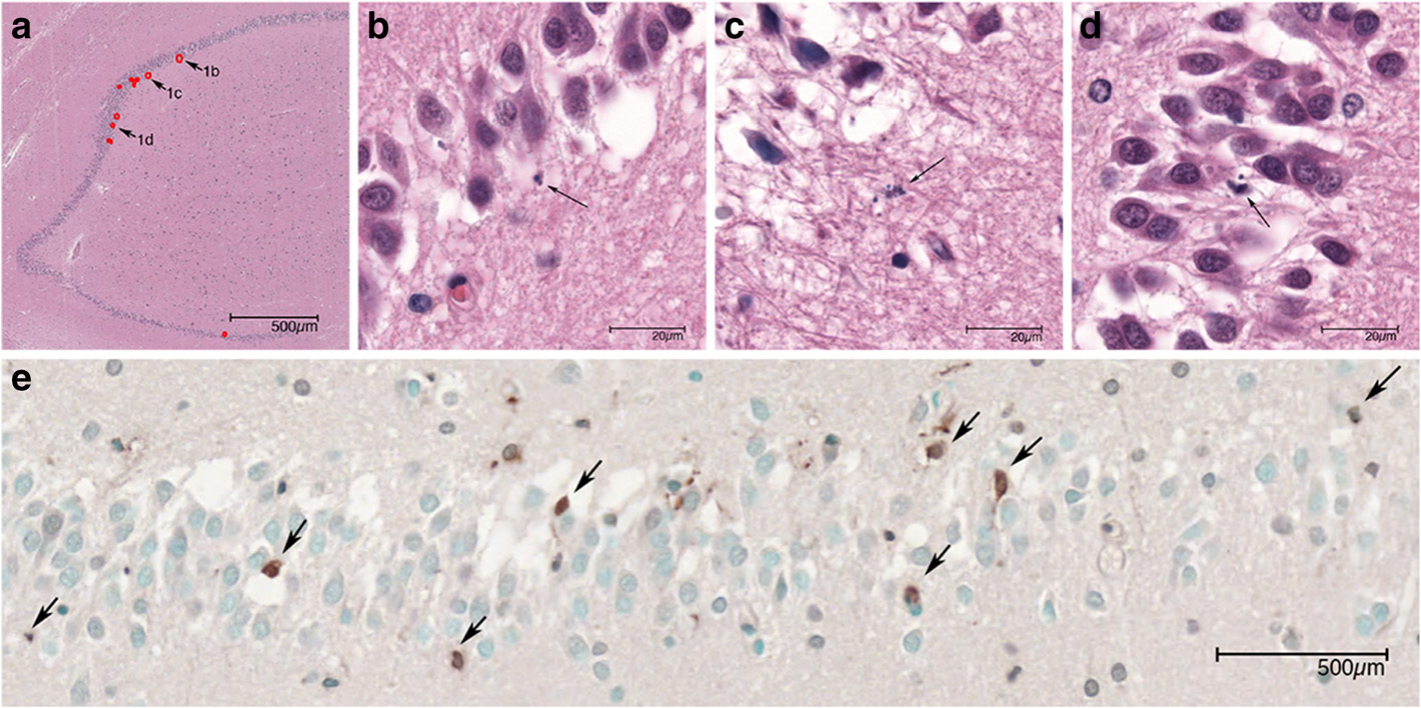
Apoptotic cells in hippocampus dentate gyrus. **a** Overview of hippocampus in HE stain. The apoptotic cells are marked with red dots. **b**–**d** Three apoptotic cells are indicated by arrows and magnified in **b**, **c** and **d**. Arrows again indicate the apoptotic cells. **e**. TUNEL analysis. Arrows indicate the positive cells

At the time of autopsy the brains were macroscopically examined by the local pathologists, followed by formalin fixation and sampling of macroscopically abnormal lesions. The sampled cut-up blocks were then embedded in paraffin, cut, deparaffinized and stained for hematoxylin-eosin (HE) at the local institutes. Other histologic and immunohistologic stainings such as Luxol-PAS, Nissl, Elastica van Gieson, a number of routine neuronal and glial marker were performed at the discretion of the pathologist. All available slides and cut-up blocks were collected at the AMC. All HE slides were re-evaluated by a neuro-pathologist and scored for the following parameters: 1) severity and main components of the inflammatory cells, 2) brain parenchymal damages consisting of cerebral infarction, haemorrhage, abscess and parenchymal infiltration of inflammatory cells, 3) inflammation in large/medium arteries and small parenchymal vessels, 4) arterial, venous and capillary thrombosis and 5) presence of ventriculitis. A Carl Zeiss Axiopskop light microscope with 6 object lenses of magnification of ×2, ×4, ×10, ×20, ×40 and ×100 and LED light source was used (Wetzlar, Germany). For the counting of apoptotic cells, the slides with hippocampus area were scanned with A. Menarini D-Sight fluo scanner (Florence, Italy) and the entire surface of hippocampal dentate gyrus were measured using the D-Sight Viewer 4.0 software (Brussels, Belgium). Apoptotic cells were identified by microscopic investigation with a 100× oil lens magnification. Apoptotic cells were distinguished by the presence of condensed chromatin with budding of nucleus and/or apoptotic bodies (Fig. 1a). If there was doubt whether cell types other than hippocampal cells, were present, such as inflammatory cells, the case was excluded for the apoptosis analysis. The number of apoptotic cells was converted to total apoptosis/mm^2^.

### TUNEL in situ assay

We used FD NeuroApop Kit (FD NeuroTechnologies) for detection of DNA fragmentation in apoptosis in the hippocampal region. Paraffin blocks were cut in 5 μm thickness and mounted on the slides, followed by deparaffinization and rehydration. After washing with PBS, the slides were incubated with 100 μl Digestive Enzyme for 15 min. The slides were then incubated with reaction buffer and then with the Detection Reagent. The slides were covered with Chromogen solution and subsequently counterstained by incubating with Methyl Green. The process was finalized with rehydration and cover slips mounting using permount. Positive stained cells (Fig. 1b) of hippocampal dentate gyrus were manually counted and the number of apoptotic cells was converted to total apoptosis/mm^2^.

### Data analysis

Five categories were defined for histological evaluation: meningeal infiltration of inflammatory cells, brain parenchymal damage, vascular inflammation, thrombosis and ventriculitis. Brain parenchymal damage was evaluated by assessing parenchymal infiltration of inflammatory cells, infarction, haemorrhage and abscesses. For the vascular inflammation, inflammation of medium-large arteries in the meninges and small parenchymal vessels were evaluated. Thrombosis was sub-categorized into thrombosis of arterial, venous and small vascular thrombosis. Histopathological findings were scored according to Table 1.

**Table 1:** Histopathological scoring of pneumococcal meningitis

Scoring criteria		Scores			
Main category	Subcategory	0	1	2	3
Meningeal infiltration		Absent	Focal mild infiltration	Multifocal mild or focal severe infiltration	Multifocal severe infiltration
Parenchymal damage	Parenchymal infiltration of inflammatory cells	Absent	Focal mild infiltration	Multifocal mild or focal severe infiltration	Multifocal severe infiltration
	Infarction	Absent	Focal small damage	Multifocal small or focal large inflammation	Multifocal large damages
	Haemorrhage				
	Abscess				
Vascular inflammation	Large meningeal artery inflammation	Absent	Focal mild (sub)endothelial infiltration/reactive changes	Multifocal mild (sub)endothelial infiltration/reactive changes or focal severe (sub)-endothelial infiltration with obstruction of vascular lumen and/or extension of infiltration in the media layer	Multifocal severe (sub)endothelial infiltration with obstruction of vascular lumen and/or extension of infiltration in the media layer
	Small parenchymal vessel inflammation				
Thrombosis	Arterial thrombosis	Absent	Focal mild with partial obstruction of vascular lumen	Multifocal mild with partial obstruction of vascular lumen or focal severe with complete obstruction of vascular lumen and destruction of vessel wall	Multifocal severe with complete obstruction of vascular lumen and destruction of vessel wall
	Venous thrombosis				
	Small vessel thrombosis				
Ventriculitis		Absent	A few inflammatory cells in the ventricle	Groups of inflammatory cells in the ventricle with/without ependymal infiltration	Extension of inflammatory cells into the periventricular tissue

Continuous data are presented as medians and interquartile ranges (IQR). Differences in pathology scores between patient groups were compared using a Mann Whitney U-test for continuous variables and a Chi-square test or Fisher’s exact test regarding dichotomous variables. Strength of relationships between continuous variables was assessed by Spearman’s correlation tests. All statistical tests were 2-tailed, and a p-value of <0.05 was considered to be significant. All analyses were executed using SPSS software, version 21.0.

## Results

### Selection of patients

**Fig. 2:**
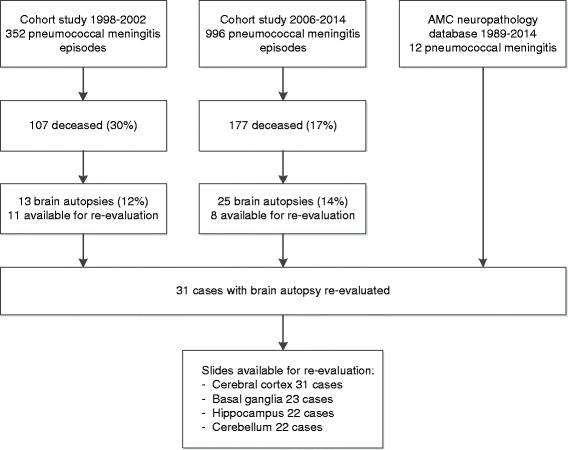
Flow chart patient selection

We identified 31 cases: 12 identified from the AMC neuropathology databank, 11 from the nationwide cohort study 1998–2002, and 8 from the nationwide cohort study 2006–2015 [2, 6, 21]. The patient selection process is described in Fig. 2. Out of 31 cases, a median number of 11 slides per case (IQR 7–14) were available and evaluated. Number of brain areas evaluated per patient are noted in Additional file 1: Table S1.

### Clinical characteristics, treatment and clinical course

Baseline characteristics were available for all patients and detailed clinical characteristics could be retrieved from 27 patients (87 %; Table 2); 22 patients were included in prospective clinical studies. The median age was 67 years (IQR 46–77), and the majority was male (64 %). Predisposing factors were present in 13 of 27 patients (48 %). All but one patient were admitted with a decreased level of consciousness, defined a score on the Glasgow Coma Scale (GCS) of 14 or lower, and 8 of 23 (35 %) were admitted in coma, as defined as a GCS score below 8. The median C-reactive protein level in blood was 303 mg/L (IQR 204–403). The median CSF leukocyte count 657 per mm^3^ (IQR 51–1787). Antibiotic treatment consisted of a third generation cephalosporin (ceftriaxone or cefotaxime) combined with amoxicillin in 7 of 23 cases (30 %), monotherapy amoxicillin or penicillin in 4 (17 %), monotherapy third generation cephalosporin in 8 (35 %), and other regimens were used in 3 (13 %) patients. Adjunctive dexamethasone was given before or with the first dose of antibiotics in 11 of 25 cases (44 %) including 10 patients (40 %) who received dexamethasone 10 mg QID for 4 days according to current guidelines [22]; 5 additional patients received dexamethasone after the first dose of antibiotics or after clinical deterioration. Baseline characteristics, clinical presentation and complications were similar between patients receiving adjunctive dexamethasone with or before the first dose of antibiotics and those who did not (Additional file 2: Table S2).

**Table 2:** Patient characteristics^a^

Characteristic	n/N (%)	Characteristic	n/N (%)
Age (years)	67 (46–77)	Indexes of inflammation in CSF^e^	
Female	11/31 (36 %)	Leukocyte count (cells/mm^3^)	656 (51–1787)
Predisposing factors	13/27 (48 %)	Protein level (g/L)	5.1 (2.4–7.2)
Otitis/sinusitis	7/25 (28 %)	CSF/blood glucose ratio	0.01 (0.00–0.15)
Pneumonia	6/24 (25 %)	Antibiotic treatment	
Immunocompromised state^b^	7/27 (26 %)	3rd gen cephalosporin + amoxicillin	7/23 (35 %)
Symptoms and signs on admission		3rd gen cephalosporin	8/23 (30 %)
Duration of symptoms >24 h	12/23 (53 %)	Amoxicillin or penicillin	4/23 (17 %)
Headache	9/17 (53 %)	Other regimens	3/23 (13 %)
Nausea	8/15 (53 %)	Adjunctive dexamethasone	
Temperature ≥38 °C	14/21 (67 %)	Started before or with first dose antibiotics	11/25 (44 %)
Neck stiffness	8/22 (36 %)	10 mg QID 4 days	10/25 (40 %)
Triad of neck stiffness, fever and altered mental status	8/22 (36 %)	Started after first dose of antibiotics	5/25 (20 %)
Seizures	8/23 (35 %)	Complications	
Score on Glasgow Coma Scale^c^	10 (7–11)	Cardiorespiratory failure	17/26 (74 %)
Altered mental state (GCS <14)	22/23 (96 %)	Mechanical ventilation	18/22 (82 %)
Coma (GCS <8)	8/22 (36 %)	Seizures	8/19 (30 %)
Focal neurological deficits		Cerebral infarction	8/18 (44 %)
Blood chemistry tests^d^		Cerebral haemorrhage	2/18 (11 %)
Leukocyte count (x10^9^/L)	15 (8–20)	Cerebral herniation	3
Thrombocyte count (x109/L)	189 (133–274)	Time to death (days)^f^	7 (3–30)
C-reactive protein (mg/L)	303 (204–403)	Early death (<7 days)	14/27
Erythrocyte sedimentation rate (mm/h, range)	51 (40–90)	Range	2–62 days

^a^Data are presented as n/N (%) or median (interquartile range) ^b^Defined as the use of immunosuppressive drugs, a history of cancer or diabetes mellitus. ^c^Glasgow Coma Scale score was known for 23 patients. ^d^Leukocyte count was known for 25 patients, thrombocyte count in 24 patients, serum C-reactive protein (CRP) levels in 15 patients, Erythrocyte sedimentation rate (ESR) in 15 patients. ^e^CSF leukocyte count was known in 23 patients, CSF protein concentration in 20 patients, CSF to blood glucose ratio in 23 patients. ^f^Time from admission to death was known for 27 patients

Neurological complications developed during clinical course in 22 of 23 patients (96 %), and systemic complications in 21 of 23 patients evaluated (91 %). A clinical diagnosis of stroke was made in 13 of 20 (65 %), and consisted of cerebral infarction in 11 (55 %) and cerebral haemorrhage in 2 (10 %). Three patients were clinically diagnosed with secondary deterioration due to multiple cerebral infarctions, consistent with the diagnosis of delayed cerebral thrombosis [25, 26]. The time from admission to death was known for 27 of 31 patients (87 %). Fourteen patients died within 7 days of admission and were considered to be in the early phase of meningitis (median duration of disease 3 days, IQR 2–4). Thirteen cases with a time to death of 7 days or longer were classified as late phase, with a median duration of disease of 27 days (IQR 16–31).

### Total pathology score

The median total pathology score was 15 (IQR 10–20; maximal score is 33; Table 3). Total pathology score was associated with age (older than 60 years, 11 [IQR 9–20] *vs.* 60 years or younger, 18 [IQR 12–21]; *p* = 0.042). None of the other clinical features or laboratory results was associated with pathology scores. However, patients treated with dexamethasone started prior to or together with the first dose of antibiotics had a higher median total pathology score (median total pathology score 18 [IQR 17–24] *vs.* 11 [IQR 7–17]; *p* = 0.003). Patients treated with dexamethasone had higher scores on parenchymal infiltration, infarction and bleeding (Additional file 2: Table S2), but not for thrombosis of arteries, veins and capillaries (data not shown). Causes of death were determined in 16 of 31 patients and listed in Additional file 3: Table S3.

**Table 3:** Summary of pathological findings^a^

	All patients	Score	Early phase^b^	Score	Late phase^b^	Score
Meningeal inflammation	31/31 (100 %)	3	14/14 (100 %)	3	13/13(100 %)	2
Infarction	19/31 (61 %)	2	5/14 (36 %)	0	11/13 (85 %)	3
Haemorrhage	24/31 (77 %)	1	9/14 (64 %)	1	11/13 (85 %)	1
Abscess	6/31 (19 %)	0	4/14 (29 %)	0	2/13 (15 %)	0
Parenchymal infiltration of inflammatory cells	24/31 (77 %)	2	10/14 (71 %)	1	10/13 (77 %)	2
Inflammation of medium large arteries in meninges	30/31 (97 %)	2	14/14 (100 %)	2	12/13 (92 %)	2
Inflammation of small parenchymal vessels	29/31 (94 %)	3	14/14 (100 %)	3	11/13 (85 %)	2
Thrombosis of medium-large arteries in meninges	15/31 (48 %)	0	4/14 (29 %)	0	9/13 (69 %)	2
Thrombosis of veins in meninges	5/31 (16 %)	0	3/14 (21 %)	0	2/13 (15 %)	0
Thrombosis of small parenchymal vessels	14/31 (45 %)	1	2/14 (15 %)	0	9/13 (69 %)	2
Ventriculitis	19/28 (68 %)	1	10/12 (83 %)	2	7/12 (58 %)	1
Total Pathology Score	31/31 (100 %)	15	14 (100 %)	14	13 (100 %)	17

^a^Data are n/N (%), or median pathology score. ^b^Early phase was defined as death <7 days, late phase >7 days

Patients treated with dexamethasone prior to or together with the first dose of antibiotics died more often because of cerebral causes than systemic causes (9 of 9 [100 %] vs. 2 of 6 [33 %]; *p* = 0.01).

### Meningeal inflammation

**Fig. 3:**
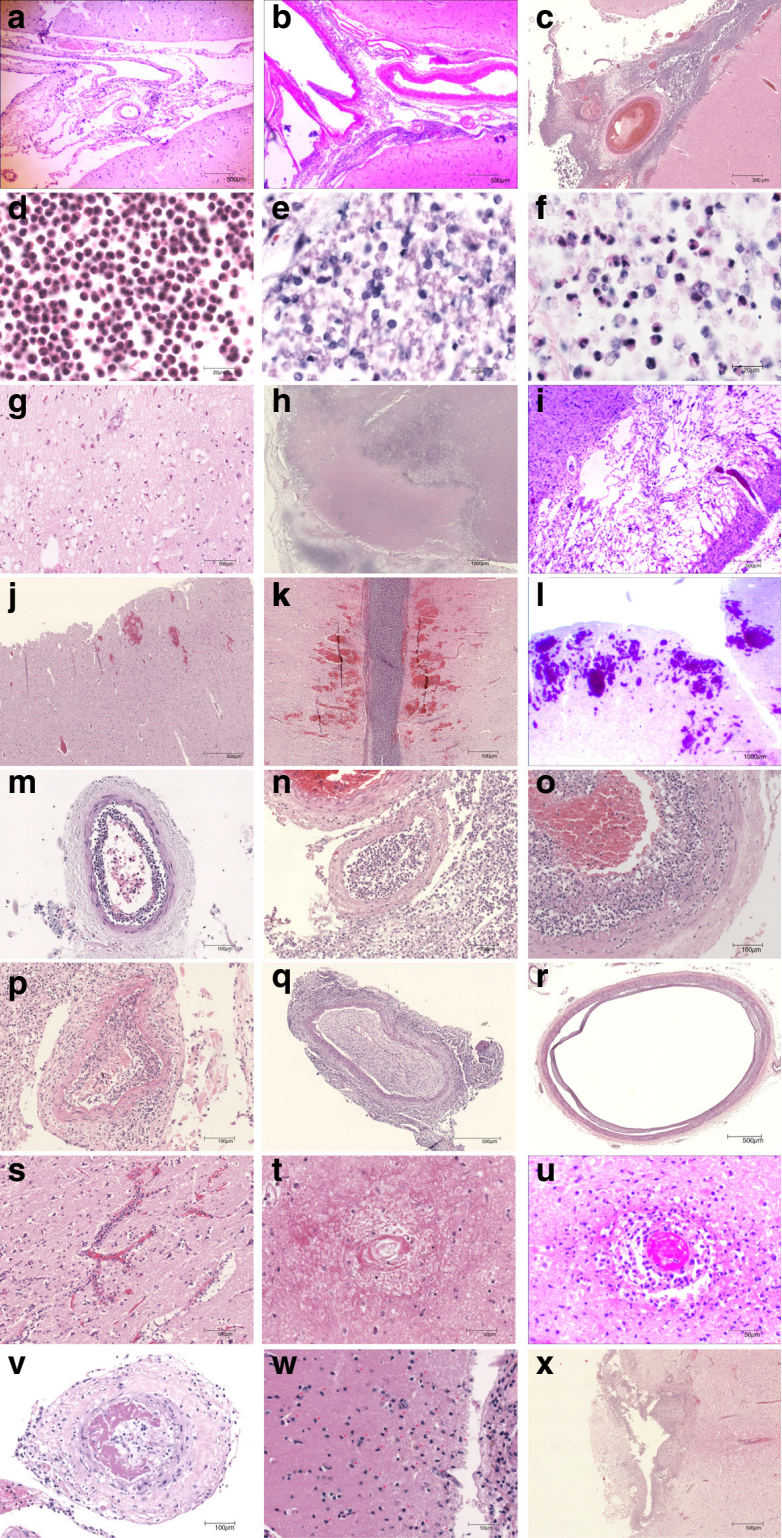
Pathological findings in pneumococcal meningitis. Short illustration of histological abnormalities. The score is based on severity of each lesion and also extensiveness/multifocality of the histological abnomalities. **a**–**c** Meningeal inflammation: Little (**a**), moderate (**b**) and extensive (**c**) infiltration of neutrophils in the sub-arachnoid space. **d**–**f** Meningeal inflammation components: mainly neutrophils (**d**), mainly macrophages (**e**) and mix of neutrophils and macrophages (**f**) in a late phase case. **g**–**i** Infarction: focal small hypoxic-ischemic neuronal injury with eosinophilic neurons (**g**), focal large established infarct with tissue degeneration and infiltration of inflammatory cells (**h**) and one of multifocal large infarcts with gliosis in the late phase (**i**). **j**–**l** Haemorrhage: focal small perivascular haemorrhages (**j**), multifocal parenchymal moderate size haemorrhages (**k**) and multifocal large haemorrhage (**l**). **m**–**o** Large-medium size meningeal arterial inflammation in the early phase: mild (m, admission day 3) and severe sub-endothelial inflammation with near obstruction of vascular lumen in n (admission day 3). In o (admission day 3), vascular inflammation extends into the tunica media with destruction of elastica layer. **p**–**r** Pathological changes of large-medium size arteries in the late phase: broadened tunica intima with formation of connective tissue infiltrated by various quantities of mixed inflammatory cell composed of macrophages, lymphocytes, plasma cells and neutrophils (**p**, admission day 18), reactive change of artery with thickening of tunica intima with near obstruction of lumen (**q**, admission day 21), and reactive change of artery with tunica media degeneration and dilation (**r**, admission day 30). **s**–**u** Pathological changes of small brain parenchymal vessels: inflammation (**s**), total destruction and degeneration (**t**) and thrombosis (**u**). **v**–**x** Thrombosis and parenchymal infiltration of inflammatory cells: thrombosis of artery with total obstruction (**v**), parenchymal infiltration of inflammatory cells (**w**, neutrophils are marked with asterick) and a small abscess in basal ganglia (**x**)

Meningeal inflammation was present in all 31 evaluated patients. Three cases (10 %) showed focal mild inflammation in the subarachnoid space (Fig. 3a), 10 (32 %) multifocal mild/focal severe/moderate (Fig. 3b) and 18 (58 %) multifocal severe meningeal inflammation (Fig. 3c). A dominant polymorphonuclear leucocytosis (Fig. 3d), defined as more than 90 % polymorphonuclear leukocytes in the subarachnoid space, was seen in 11 of 14 early phase patients (79 %). Over time, increasing with the days between admission and death, the proportion of polymorphonuclear leukoyctes decreased; late cases showed monocyte dominancy with an estimated macrophage ratio of 80–90 % (in 10 of 13 patients [77 %], Fig. 3e). However, three late phase patients showed a persistent polymorphonuclear leukocytosis of more than 50 % despite a duration between admission and death of 10, 30 and 31 days (Fig. 3f).

### Brain parenchymal damage

Parenchymal infiltration of inflammatory cells (cerebritis, Fig. 3w) was seen in 24 of 31 cases (77 %); six cases (19 %) with focal mild infiltration, 12 (38 %) with multifocal mild or focal severe and 6 (19 %) with extensive infiltration, distributed over similar proportions for early and late phase patients (10 of 14 [71 %] *vs.* 10 of 13 [77 %]; *p* = 0.74). Direct infiltration of inflammatory cells in the brain parenchyma usually was limited to the superficial part of cortex. Extensive parenchymal infiltration of inflammatory cells occurred primarily at the site of parenchymal tissue damage, for instance in areas of infarction, haemorrhage or abscess formation.

Cerebral infarction was identified in 19 patients (61 %); 2 cases (6 %) with focal small infarction (Fig. 3g), 4 (13 %) with multifocal small/focal large infarction (Fig. 3h) and 13 (42 %) with extensive infarction (Fig. 3i). Cerebral haemorrhage was seen in 24 patients (77 %); 13 (42 %) with focal small haemorrhage (Fig. 3j), 3 (10 %) with multifocal small/focal large haemorrhages (Fig. 3k) and 8 (26 %) cases with multifocal large haemorrhages (Fig. 3l). 17 patients (55 %) had both cerebral infarction and haemorrhage. Only 5 patients (16 %) had neither infarction nor bleeding. Infarction was more frequently observed in the late phase than in the early phases (11 of 13 patients [85 %] *vs.* 5 of 14 patients [36 %], *p* = 0.001), while presence of haemorrhage was not related to disease duration (11 of 13 patients in the late phase [85 %] *vs.* 9 of 14 patients in the early phase [64 %], *p* = 0.23). Patients without infarction or haemorrhage all died early (<5 days). Cranial CT or MRI was performed in 20 of 27 (74 %) patients upon admission and in 16 patients (59 %) during admission, and showed cerebral haemorrhage in two patients and cerebral infarction in nine patients. Cerebrovascular complications on cranial imaging were not significantly associated with cerebral infarctions or haemorrhages observed in the pathological analysis. Whether cranial imaging was performed and timing between admission, cranial imaging and death varied considerably between patients which may have contributed to the lack of radiological-pathological correlation.

Brain abscess (Fig. 3x) was present in 6 of 31 cases (19 %), and all were focal and small: 4 patients in early phase and 2 in late phase cases. Identified abscesses were small with a median diameter of 3 mm (range 1-6 mm). Clinical data were available for 5 of patients with abscess formation; one died on hospital arrival prior to treatment or investigations. None of the other 4 patients were diagnosed with brain abscess prior to autopsy.

### Vascular inflammation

Various degrees of inflammation of large-medium arteries and the consequent reactive changes were seen in 30 of 31 patients (97 %). The early phase patients showed sub-endothelial influx of neutrophils in the arteries with endothelial damage. The subendothelial accumulation of neutrophils (subendothelial pus) can be mild with open lumen (Fig. 3m). In 6 of 14 early phase cases (43 %), however, a severe narrowing of the vascular lumen was seen by the subendothelial pus (Fig. 3n), of which 5 cases had complete obstruction of arteries. The vascular inflammation extended into the tunica media with destruction of tunica elastica layer (Fig. 3o) in 3 of 14 early phase patients (21 %). Vascular inflammation in late phase cases was characterized by reactive changes, represented as broadened tunica intima with formation of connective tissue infiltrated by various quantities of mixed inflammatory cell composed of macrophages, lymphocytes, plasma cells and neutrophils, as a result narrowed vascular lumen (Fig. 3p). These reactive changes in vascular inflammation led to severe narrowing of the arterial lumen in 8 of 13 cases (62 %). Presence of complete obstruction of arteries was seen in 7 of 13 cases ([54 %], Fig. 3q). In one late phase case, dilatation of arteries was seen as a result of destruction of tunica media (Fig. 3r). Inflammation of small vessels was observed in 29 of 31 cases (94 %, Fig. 3s); five patients (16 %) with focal mild, 8 (26 %) with multifocal mild or focal severe and 16 (52 %) with multifocal severe small vascular inflammation. In 9 of 13 late cases (69 %), the inflamed small vessels were destructed and occluded with connective tissue reaction (endarteritis obliterans, Fig. 3t).

Presence of vascular inflammation was associated with the clinical diagnosis of infarction: clinical diagnosed cerebral infarction was observed in 2 of 12 (17 %) cases without vascular inflammation compared to 12 of 19 (63 %) cases with vascular inflammation (*p* = 0.01). Arterial thrombosis was associated with co-existing capillary thrombosis (capillary thrombosis present in 13 of 16 with arterial thrombosis *vs.* 2 of 15 without arterial thrombosis; *p* = 0.002).

### Thrombosis and ventriculitis

Thrombosis, thrombo-embolism and/or sinus thrombosis was observed in 21 of 31 patients (68 %). Thrombosis of arteries (Fig. 3v) was more frequently observed in the late phase than the early phase (9 of 13 late phase cases [69 %] *vs.* 4 of 14 early phase cases [29 %]; *p* = 0.04). Thrombosis of parenchymal small vessels (Fig. 3t) was also more frequently seen in the late phase (9 of 13 late phase cases [69 %] *vs.* 2 of 14 early phase cases [15 %], *p* = 0.004). Venous thrombosis was found in 3 of 14 patients in the early phase (21 %) *vs.* 2 of 13 patients in the late phase (15 %; *p* = 0.69). The ventricle was sampled in 28 cases; 12 early phase cases, 12 late phase cases and 4 with unknown duration of disease. Ventriculitis was present in 19 patients (68 %), 10 of 12 patients in the early phase (83 %) and 7 of 12 patients in the late phases (58 %), *p* = 0.18. Seven cases showed focal mild ventriculitis (2 of 12 early phase cases [17 %] *vs.* 5 of 12 late phase cases [42 %], *p* = 0.18) and 9 cases were with more extensive inflammation, but still limited in the ependyma. More severe inflammation with periventricular involvement of the inflammation was seen in two patients, both in the early phase.

### Apoptosis of hippocampal dentate gyrus

Of the 31 meningitis cases, HE staining of hippocampus was available for 21 patients (median age 68 years [IQR 46–76]; male-female ratio of 13:8). The time between admission and death was known in 17 cases (median, 8 days [IQR 2–20]). There were 21 control non-meningitis cases (median age 70 year [IQR 53–77]; male-female ratio of 13:8). For controls the median days between hospital admission and death was 7 days (IQR 2–14). The median hippocampal dentate gyrus surface evaluated was 0.81 mm^2^ for cases (IQR 0.66–0.93 mm^2^) and median 0.65 mm^2^ (IQR 0.54–0.73 mm^2^) for controls. Morphologic evaluation of apoptotic cells in HE staining showed a median of 3 apoptotic cells (IQR 1–9) per mm^2^ in cases and 0 apoptotic cells (IQR 0–7) per mm^2^ in controls.

For TUNEL assay paraffin blocks of hippocampus were available for 13 patients with pneumococcal meningitis and 20 controls; these samples were used for morphologic analysis in HE staining. The median total surface of the hippocampal dentate gyrus was 0.76 mm^2^ (IQR 0.66–0.90 mm^2^) for meningitis patients and 0.66 mm^2^ (IQR 0.56–0.73 mm^2^) for controls. There was no significant difference seen in the number of apoptotic cells between the case and control groups, neither in morphologic analysis in HE staining (median 3/mm^2^ in patients vs. 0/mm^2^, in controls *p* = 0.12) or in TUNEL assay (median 2/mm^2^ in patients vs. 7/mm^2^, in controls *p* = 0.19).

Information on adjunctive dexamethasone therapy was available for 16 of 21 patients with morphologic analysis of HE staining: 11 received dexamethasone and 5 did not. The median number of apoptotic cells was similar between groups (5/mm^2^*vs.* 3/mm^2^; *p* = 0.66). There were no associations between the total number of apoptotic cells and the total surface of dentate gyrus, age, the interval from onset of symptoms to death and the pathology scores (data not shown).

## Discussion

Our study shows that vascular damage is key in the process of brain damage in pneumococcal meningitis. All patients had medium-large artery inflammation, cerebral haemorrhage, and infarction. Gross changes, such as pressure coming, which have been described as an obvious cause of death in pneumococcal meningitis, were rarely found. Previous autopsy studies showed inflammatory infiltration of cerebral veins and arteries [27–29], but did not correlate these findings with clinical data. In the current study we showed that in early disease phase, severe narrowing of the vascular lumen was mainly caused by subendothelial pus with subsequent haemorrhages. In the late phase of disease, after seven days of admission, the narrowed vascular lumen was characterized by reactive vascular changes, represented as broadened tunica intima with formation of connective tissue infiltrated by various quantities of mixed inflammatory cells.

We did not observe extensive parenchymal leukocyte infiltration. Extensive infiltration of inflammatory cells occurred primarily at the site of parenchymal tissue damage, for instance in areas of infarction, haemorrhage or abscess formation. This finding is consistent with previous experimental studies, showing that leukocyte infiltration into the brain parenchyma has only been observed during late infection and in the direct vicinity of the fluid-filled spaces [17, 30]. Other elements previously described leading to permanent brain injury such as cortical necrosis, cerebral oedema, hydrocephalus, hippocampal apoptosis do occur but seem to be less essential as previously thought [9, 11, 23, 31]. Our data suggest that future studies should focus on generalized dampening of the inflammatory response in the very early phase of disease [23], for example using adjunctive dexamethasone therapy, complement inhibition [23, 32, 33], or target the pathological changes in the brain vasculature.

Vascular inflammation can be divided in early and late phase characteristics. We observed inflammation of medium large arteries in meninges in the early phase with endothelial swelling and/or ulceration, inflammation and necrosis of tunica media and thrombus forming. This finding is consistent with previous reports [8, 28]. Systematic analysis of the frequency and severity of the vascular inflammation showed that all patients exhibit arterial inflammation, with mild narrowing of lumen to (near) obstruction, resulting in vascular obstruction in the majority of patients. This severe narrowing and obstruction of the arterial lumen is an important mechanism of brain parenchymal infarction in pneumococcal meningitis, especially in the early phase where thrombosis plays a less important role. This is in line with a retrospective transcranial doppler study of in 94 patients with acute bacterial meningitis, showing increased cerebral blood flow velocity in about half of patients, which was associated with increased risk of cerebral infarction (odds ratio 9.15; 95 % confidence interval 1.96–42.67) [34]. The ongoing reactive changes of medium large arteries, which again causes narrowing and obstruction of the vascular lumen, as well as thrombus formation in the arteries and small vessels, are likely to be responsible for the increased frequency and severity of infarctions in the late phase.

The pathogenesis of cerebral infarction is a subject of ongoing research, which has focused largely on two areas: first, the dysregulation of the coagulation and fibrinolysis [35, 36], not only systemically but also locally [37, 38], as exemplified by the upregulation of plasminogen activator inhibitor-1 and elevated levels of prothrombin fragments F1 and −2 and soluble tissue factor in the CSF of patients with pneumococcal meningitis [36]; and second, endothelial cell dysfunction, which may lead to localized swelling and release of pro-coagulant factors and proinflammatory cytokines [39–41]. Our data suggest an important role for cerebral (micro)haemorrhages. Although speculative, we hypothesize that in early phases, bacteria indeed adhere to blood vessel endothelium of the blood-brain-barrier using complex mechanisms such as binding of endothelial laminin receptor, PAF receptors and intercellular translocation [23]. Subsequently, bacteria may well bluntly disrupt and invade the basement membrane, causing (micro)hemorrhages, which may well result in secondary cerebral infarction through vessel thrombosis, vasospams, and loss of cerebral autoregulation. Alternatively, massive clotting may also result in local depletion of clotting factors, thereby introducing micro-hemorrhages [23]. Few studies addressed visualization of bacterial growth and spread to the central nervous system from blood by breaching the multiple layers of meninges and brain-blood barrier. Many questions remains unanswered, such as how the bacteria invade the brain parenchyma and how bacteria cause the large and small vascular inflammation.

We found that adjunctive dexamethasone treatment started prior to or with the first dose of antibiotics was not associated with increased hippocampal apoptosis. Cognitive impairment is a frequent complication in adults who survive bacterial meningitis [42]. Because corticosteroids may potentiate ischemic injury to neurons [43], it is important to know whether dexamethasone prevents death but worsens cerebral functioning. An experimental pneumococcal meningitis study identified aggravated apoptosis in the hippocampus of infant rats treated with dexamethasone, which was associated with learning deficiency [11]. We did not detect any differences in the number of apoptotic cell in the hippocampal dentate gyrus using HE and TUNEL between patients treated with or without dexamethasone. Furthermore, we did not find any differences in dentate gyrus apoptosis between meningitis patients and controls. This is in contrast with a previous study showing that described hippocampal apoptosis demonstrated by HE and TUNEL assay in 26 brains from bacterial meningitis patients of whom nine had pneumococcal meningitis [9]. Results of our and this study are, however, difficult to compared due to differences in analyses and included populations. Our results imply the role of hippocampal apoptosis is limited in pneumococcal meningitis, and are in line with a follow-up study of the European Dexamethasone Study that described that treatment with adjunctive dexamethasone is not associated with an increased risk for long-term cognitive impairment [6].

Adjunctive dexamethasone treatment was associated with more brain pathology. Brains of the patients treated with dexamethasone showed more infarctions, haemorrhages, arterial inflammation and parenchymal infiltration of inflammatory cells. Patients treated with dexamethasone all died due to neurological causes whereas the majority of non-dexamethasone treated patients died due to systemic causes. This is in line with the finding that the beneficial effect of dexamethasone on mortality rates in patients with pneumococcal meningitis is attributable to a beneficial effect on systemic complications [44].

Our study has several limitations. First, autopsy is only performed in approximately 20 % of deceased patients in our cohort studies and brain autopsy was not performed in all of them. For both cohorts together we were able to re-evaluate brains of 5 % of deceased patients [2, 22]. Although this is only a small proportion of potential cases, it is unlikely the sample has a selection bias that would decrease the value of the analysis. Second, we were also limited to microscopic investigation of different quantity of slides from varying brain regions. Conclusions of pathology analysis and clinical-pathological correlations were based on a limited number of observations (slides) per brain and therefore may suffer from sampling error. Third, several patients were identified retrospectively from the AMC neuropathology database, and clinical data could not or only partially be retrieved for some of them. This resulted in missing data on duration of disease in four patients that may therefore potentially have died from different causes than meningitis. However, in these patients the autopsy reports did not indicate a different cause of death. Fourth, we did not have data on onset of therapy or exact duration of disease before admission, which, if unevenly distributed between dexamethasone groups, may be a confounding factor. Finally, to what extent the described pathology processes occur in patients surviving pneumococcal meningitis cannot be determined from our study. Nevertheless, our series is currently the largest collection of pathological analysis with clinical data, and provides valuable leads for further pathophysiological studies. This work is part of a prospective cohort study to identify and characterise host genetic traits and bacterial genetic factors controlling occurrence and outcome of bacterial meningitis (MeninGene) [21, 24]. We are currently working on digitalization of our pathology material, which will be made publically accessible (MeninGene-Path). Specimens will also be made available for further imaging and expression studies.

## Conclusion

Vascular inflammation plays an important role in brain damage of pneumococcal meningitis throughout the whole disease process, from the early to late phases. Dexamethasone was not shown to have significant influence in hippocampal apoptosis, Brains of the patients treated with dexamethasone showed however more pathological changes, which is probably attributable to the beneficial effect of dexamethasone on mortality rates in patients with pneumococcal meningitis as a result of its beneficial effect on systemic complications.

## Additional files

Additional file 1: Table S1. Available histopathology slides per brain area. (DOC 31 kb) Additional file 2: Table S2. Summary of statistical analysis of pathological findings in relation to clinical characteristics. (DOC 42 kb) Additional file 3: Table S3. Cause of death in Dexamethasone treatment and non-dexamethasone treatment groups. (DOC 34 kb)
