# Supplementary material for: Pneumococcal meningitis: Clinical-pathological correlations (meningene-path)
Source: Acta Neuropathol Commun. 2016 Mar 22;4:26. doi: 10.1186/s40478-016-0297-4 (PMC4802600; doi:10.1186/s40478-016-0297-4)
Supplement: Additional file 1: Table S1. — Available histopathology slides per brain area. (DOC 31 kb) [file 40478_2016_297_MOESM1_ESM.doc]

**Table S1.** Available histopathology slides per brain area.

| Sampled areas | No of cases (percentage) | Median  (Range) |
| --- | --- | --- |
| Cortex | 31 (100%) | 4 (1-11) |
| Basal ganglia | 23 (74%) | 2 (0-5) |
| Cerebellum | 22 (71%) | 1 (0-4) |
| Hippocampus | 21 (68%) | 1 (0-4) |
| Mesencephalon | 19 (61%) | 1 (0-2) |
| Pons | 18 (58%) | 1 (0-4) |
| Medulla oblongata | 17 (55%) | 0,5 (0-2) |
| Spinal cord | 4 (13%) | 0 (0-3) |
| Separated arachnoid | 5 (16%) | 0 (0-1) |
| Sinus sagittalis | 4 (13%) | 0 (0-3) |
| Separated sample large vessels | 1 (3%) | 0 (0-1) |
