# Supplementary material for: Pneumococcal meningitis: Clinical-pathological correlations (meningene-path)
Source: Acta Neuropathol Commun. 2016 Mar 22;4:26. doi: 10.1186/s40478-016-0297-4 (PMC4802600; doi:10.1186/s40478-016-0297-4)
Supplement: Additional file 2: Table S2. — Summary of statistical analysis of pathological findings in relation to clinical characteristics. (DOC 42 kb) [file 40478_2016_297_MOESM2_ESM.doc]

**Table S2.** Summary of statistical analysis of pathological findings in relation to clinical characteristics

| Characteristics | Dexamethasone with or before antibiotics (n=11) | No dexamethasone or inappropriate dose or timing (n=14) | P-value |
| --- | --- | --- | --- |
| Age (years) | 58 (46-73) | 69 (45-78) | 0.57 |
| Female | 4/11 (36%) | 5/14 (36%) | 1.00 |
| Immunocompromised state | 3/10 (30%) | 2/14 (14%) | 0.61 |
| Temperature | 37.9 (36.7-39.0) | 38.8 (37.0-40.0) | 0.41 |
| Score on Glasgow Coma Scale | 9 (4-11) | 10 (8-12) | 0.28 |
| Blood chemistry tests |  |  |  |
| Leukocyte count (x10^9^/L) | 11.1 (7.5-20.3) | 15.6 (9.7-18.5) | 0.78 |
| Thrombocyte count | 197 (138-273) | 184 (64-318) | 0.63 |
| C-reactive protein (mg/L) | 303 (225-413) | 261 (122-400) | 0.61 |
| Indexes of inflammation in CSF^e^ |  |  |  |
| Leukocyte count (cells/mm^3^) | 690 (45-3058) | 657 (50-1312) | 0.81 |
| Protein (g/L) | 5.1 (1.5-7.1) | 5.1 (2.6-7.5) | 0.76 |
| CSF/blood glucose ratio | 0.00 (0.00-0.02) | 0.08 (0.00-0.17) | 0.15 |
| Complications |  |  |  |
| Circulatory shock | 3/10 (30%) | 5/12 (42%) | 0.67 |
| Mechanical ventilation | 9/10 (90%) | 9/12 (75%) | 0.59 |
| Seizures | 1/10 (10%) | 4/10 (40%) | 0.30 |
| Cerebral infarction (imaging) | 3/10 (30%) | 5/9 (55%) | 1.00 |
| Cerebral haemorrhage (imaging) | 2/10 (20%) | 0/9 (0%) | 0.47 |
| Time to death (days) | 7 (3-21) | 4 (2-26) | 0.74 |
| Total pathology score (Range 0-30) | 18 (IQR 17-24) | 11 (IQR 7-17) | 0.003 |
| - Infarction (Score 0-3) | 3 (2-3) | 0 (0-3) | 0.033 |
| - Medium/ large arterial inflammation (Score 0-3) | 3 (2-3) | 2 (1-3) | 0.12 |
| - Bleeding (Score 0-3) | 3 (1-3) | 1 (0-1) | 0.011 |
