# Supplementary material for: Pneumococcal meningitis: Clinical-pathological correlations (meningene-path)
Source: Acta Neuropathol Commun. 2016 Mar 22;4:26. doi: 10.1186/s40478-016-0297-4 (PMC4802600; doi:10.1186/s40478-016-0297-4)
Supplement: Additional file 3: Table S3. — Cause of death in Dexamethasone treatment and non-dexamethasone treatment groups. (DOC 34 kb) [file 40478_2016_297_MOESM3_ESM.doc]

**Table S3.** Cause of death in Dexamethasone treatment and non-dexamethasone treatment groups.

| **Patients treated with Dexamethasone (n=9)** | **Patients not with Dexamethasone (n=7)** |
| --- | --- |
| 1. Multiple cerebral infarctions, suspected for delayed cerebral thrombosis. | 1. Withdrawal of care because of severe neurologic damage |
| 1. Brain herniation, brain death | 1. Cardiac arrest |
| 1. Brain death | 1. Dead on arrival |
| 1. Delayed cerebral thrombosis | 1. Pneumonia and poor neurologic prognosis |
| 1. Discontinued treatment by failure to improve | 1. Pneumosepsis, poor prognosis |
| 1. Diffuse vasculitis | 1. Sepsis |
| 1. Brain herniation | 1. Withdrawal of care because of severe neurologic damage |
| 1. Brain death |  |
| 1. Brain death |  |
